# IoT-Based Intervention and Home Support to Address Frailty-Related Vulnerability and Well-Being in Older Adults Living in Rural Areas

**DOI:** 10.3390/s26030975

**Published:** 2026-02-02

**Authors:** Jessica Fernández-Solana, Rodrigo Vélez-Santamaría, Ana I. Sánchez-Iglesias, Maria Isabel Villanueva-Alameda, Jerónimo J. González-Bernal, Josefa González-Santos

**Affiliations:** Department of Health Sciences, University of Burgos, 09001 Burgos, Spain; jfsolana@ubu.es (J.F.-S.);

**Keywords:** IoT technology, Silver Caregiver, sensor technologies, healthcare monitoring, Rural Health Services, older adults, quality of life, health status

## Abstract

Background: Spain has an increasingly aging population in rural areas. These individuals often face the burden of illness and the limitations it causes in solitude, leading to greater impacts on their health and quality of life. Therefore, the aim of this study was to evaluate the effectiveness of a combined IoT-based home monitoring and Silver Caregiver support intervention on health-related quality of life and functional, cognitive, emotional, and social outcomes in older adults living alone in rural settings. Material and methods: A longitudinal study was conducted with a sample of 144 older adults from rural areas who received home support through a Silver Caregiver and IoT technology. Results: Statistically significant differences were observed in cognitive status, anxiety, depression, family functionality, social support, life satisfaction, and quality of life. Conclusions: The findings indicate that the combined intervention primarily enhances psychological well-being, social connectedness, and perceived quality of life while contributing to the maintenance of basic physical function in older adults living in rural areas.

## 1. Introduction

In recent decades, life expectancy has experienced remarkable growth. This achievement, although positive, brings with it a new challenge for societies: population aging. As noted in [1], this phenomenon generates a series of challenges that we must face. Aging can be defined as “a dynamic, gradual, natural, and inevitable process that develops in the biological, psychological, and social aspects of individuals and is structured around time” [2,3]. Likewise, when discussing the phenomenon of population aging, the importance of addressing this issue within the health sector is emphasized, since it is our responsibility to meet the new needs driven by demographic dynamics and the increase in demand for health services among the elderly population [4,5,6]. According to the World Health Organization (WHO) bulletin on aging, by the year 2050, there will be more people over 65 years of age than children under 14 years of age [7].

From a biological perspective, aging occurs as a result of the progressive accumulation of various types of molecular and cellular damage over time. This leads to a gradual decline in physical and mental capacities, an increased risk of disease, and ultimately, death [8,9]. Furthermore, as populations age, the prevalence of multimorbidity increases substantially, making comorbid conditions a major determinant of mortality and functional decline in older adults [10]. Moreover, in the context of Spain, the rural environment covers 90% of the territory and is home to approximately 20% of the older population [11]. In 2019, Castilla y León was the second autonomous community to record the highest percentage of people over 65 years of age, with 25.3% [12]. Thus, it is expected that rural areas will be predominantly populated by older people. Therefore, the autonomous communities of Spain with a more aged population also tend to be the most rural, as in the case of Castilla y León, which is characterized by smaller population centers [13], a phenomenon known as the “empty Spain” [14].

Current transformations are resulting in many older people becoming disconnected from certain services. Several studies have highlighted specific characteristics of elder care in rural settings, such as difficulties in accessing hospital systems, social centers, or day centers. This is due to the fact that the network of social and health services and public support is less robust in rural areas compared to urban ones [15]. Therefore, one of the main objectives of public health today is to promote and maintain the health and well-being of an increasingly diverse aging population. In formulating a public health response to aging, the WHO [16] points out that it is important to consider not only the factors that mitigate losses associated with old age but also those that can strengthen recovery, adaptation, and psychosocial growth. In other words, the aim is to maintain, strengthen, and/or compensate for the loss of independence in life.

It is important to note that the burden of disease among the older adult population is significant due to the presence of chronic non-communicable diseases, with a marked increase observed in the burden of mental illnesses as a result of the various changes and adaptations older adults must face at this stage of life. Among the recognized changes in older people is the feeling of loneliness, which is considered a predictor of anxiety and depression, as well as the development of other health conditions [17,18,19,20].

The consequences of loneliness, or the fact that these older adults live alone, can be broken down into physical, psychological, and social dimensions. In the physical dimension, alterations may arise in their functional and nutritional status, limitations in carrying out activities of daily living (ADLs) or instrumental activities of daily living (IADLs), balance and gait problems that lead to falls, or difficulties regarding their autonomy and independence, which may translate into a risk of disability and frailty in older people. In the psychological dimension, anxiety or depression, among others, may be experienced. Meanwhile, in the social dimension, social prejudice and feelings of isolation related to a lack of family or social support may emerge [21,22]. All of this directly impacts the quality of life (QoL) of these individuals and their life satisfaction. Loneliness tends to increase among older adults who are not institutionalized as they advance in age [23]. In this context, it becomes crucial to develop monitoring programs and interventions for the prevention of loneliness and the maintenance of health in this population group [24].

The existence of information and communication technologies (ICTs) offers older adults the opportunity to enhance and improve their development both individually and socially, serving as a means to promote their autonomy and to support prevention and care, thus contributing to improving their QoL in technical, economic, political, and cultural aspects [25,26]. As evidenced by previous research [27,28], assistive technologies and new technologies play a significant role in this regard. However, due to the constant change, creation, and updating of technology, there is a need to conduct this study using Internet of Things (IoT) technology [29,30].

IoT technology refers to the grouping and interconnection of various devices and objects through an internet network, where people can connect, interact, and exchange data. These objects may range from sensors to mechanical devices embedded in everyday items, such as doors, refrigerators, footwear, and beds. This enables seamless communication between people with minimal human intervention, with technology itself responsible for collecting data, recording, monitoring, or adjusting each interaction among connected things, directly linking the physical and digital worlds and facilitating their cooperation [31,32]. The use of IoT devices for home monitoring in older adults has been shown to improve the early detection of health problems and enhance autonomy, especially in vulnerable and rural settings [33]. Likewise, digital health interventions such as mobile applications and sensors facilitate prevention and personalized care in aging, showing great potential to reduce access gaps in remote areas [34]. The use of telemedicine significantly increases access and continuity of care in rural areas, helping to overcome geographical barriers and improving the quality and equity of health services [35].

Therefore, the aim of this study was to evaluate the effectiveness of a combined intervention based on IoT-enabled home monitoring and personalized support provided by trained Silver Caregivers on health-related quality of life, functional status, cognitive performance, emotional well-being, and social support in older adults living alone in rural areas. Specifically, this study sought to examine whether the integration of continuous sensor-based monitoring with regular caregiver accompaniment and supervision could contribute to maintaining autonomy, improving safety at home, and enhancing overall well-being during daily living in this population.

## 2. Materials and Methods

### 2.1. Study Design and Participants

This longitudinal study was carried out by the University of Burgos (UBU) in collaboration with the Provincial Council of Zamora, the Regional Government of Castilla y León, and the Zamora Silver Economy Territory. The sample included 144 older adults living in rural areas. The participants were recruited through local community centers, primary care units, and senior associations in the Provincial Council of Zamora. Recruitment was conducted via informational sessions and coordination with healthcare professionals who identified eligible individuals meeting the inclusion criteria. The sample was therefore based on voluntary participation and convenience selection; although it reflects the demographic profile of the older population in the region, it cannot be considered fully representative.

The inclusion criteria were as follows: (1) dependent users from the Tierra de Campos–Pan–Lampreana area; (2) signing of informed consent; (3) absence of cognitive impairment (the absence of cognitive impairment was verified using the Mini-Mental State Examination (MMSE), and only participants who scored above 24 points, indicating normal cognitive status without cognitive impairment, were included in the study); (4) autonomy to carry out ADLs; and (5) living alone with/without family support. As exclusion criteria, all participants who did not sign the informed consent and those with total or severe dependence, measured using the Barthel Index, were excluded. The inclusion and exclusion criteria were applied consecutively until the required sample size was reached, ensuring representativeness of older adults living independently in rural areas. Screening was conducted by social and health professionals of the collaborating institutions using standardized evaluation forms to verify eligibility.

### 2.2. Procedure

For the collection of the sample in this study, the Regional Government of Castilla y León first selected, during 2022, the unique project entitled “Silver Caregivers and Rural Innovation in Elderly Care Contexts”, developed by the Provincial Council of Zamora. This project was communicated to the Ministry for the Ecological Transition and the Demographic Challenge, as stated in the certificate issued by the General Secretariat for the Demographic Challenge of that Ministry. The purpose of this project was to contribute to the sustainable development of the Tierra de Campos–Pan–Lampreana area (Zamora) through the implementation of a productive and innovative ecosystem based on the care economy and digitalization, consolidating the objectives of the Silver Economy strategy in each of the municipalities within the region.

Likewise, an innovative plan was launched to promote and strengthen the role of the “Silver Caregiver,” a professional who fosters autonomy and supports the permanence of older adults in their homes through a process of preventive accompaniment and monitoring of their routines in their everyday environment, in coordination with other agents involved in care and attention.

The aim, therefore, was to stimulate specialized and high-quality care for elderly and dependent residents, respond to cases of isolation and loneliness, foster autonomy at advanced ages, support a longer stay in their homes, and safeguard their organic, functional, cognitive, emotional, and material well-being while promoting social inclusion, self-determination, respect for their rights, and personal development. This project encompasses all these factors, digitalizing the care and attention provided to older adults through ICT solutions (such as remote care based on IoT technology and intelligent systems for monitoring home routines), with special emphasis on the “Silver Caregivers” as the main actors in the implementation of these new techniques.

The data collection process was standardized across all municipalities. All assessors received prior training from the research team to ensure uniform application of the evaluation instruments and consistency in data recording. Data were collected during home visits at baseline and after six months of intervention. All questionnaires were administered in person by the Silver Caregiver or a trained researcher, following standardized interview protocols to minimize measurement bias and ensure comparability across assessments.

The municipalities involved were Almaraz de Duero, Belver de los Montes, Benegiles, Cañizo, Castronuevo, Coreses, Manganeses de la Lampreana, Molacillos, Monfarracinos, Roales, San Cebrián de Castro, Valcabado, Villafáfila, Villanueva del Campo, Villarín de Campos, and Villaseco del Pan (Zamora, Spain).

This research was designed as a longitudinal pre–post intervention study conducted over a defined follow-up period. The study was structured into three main stages in order to systematically assess changes over time associated with the combined IoT-based monitoring and Silver Caregiver intervention.

In the first stage (baseline assessment), participants were evaluated in their homes prior to the implementation of the intervention. Sociodemographic data, clinical characteristics, comorbidity, functional status, cognitive performance, emotional state, social support, family functionality, life satisfaction, and health-related quality of life were collected using standardized assessment instruments.

The second stage corresponded to the intervention phase, during which participants received continuous home monitoring through IoT sensor technologies combined with personalized support provided by trained Silver Caregivers. This phase included remote monitoring of daily routines, detection of risk situations, regular in-person visits, emotional support, and assistance with daily activities, according to individual needs.

The third stage (post-intervention assessment) was conducted after approximately three months of intervention. During this phase, the same assessment protocol used at baseline was reapplied in order to evaluate changes in the monitored variables over time and to determine the impact of the intervention on participants’ health, autonomy, and well-being.

Data collection was carried out by designated personnel, and the data were anonymized before being shared with the research team, remaining anonymous and aggregated from that moment onward. Informed consent was obtained from the study participants, ensuring voluntariness and anonymity. Data processing was carried out in compliance with the European Data Protection Regulation and Organic Law 3/2018 on the Protection of Personal Data and the Guarantee of Digital Rights. The study protocol received approval from the Ethics Committee of the University of Burgos (Reference: UBU IR 29/2019), in accordance with the Declaration of Helsinki. All participants provided written informed consent prior to participation.

The minimum sample size required was estimated using G*Power 3.1 software, considering an expected medium effect size (f = 0.25), a statistical power of 0.80, and an alpha level of 0.05 for repeated-measures analyses. The calculation indicated a minimum of 128 participants; thus, the final sample of 144 exceeded this requirement. For ANCOVA, assuming a mean effect size (f = 0.30), a significance level of 0.05, and a power of 0.80, the required sample size was approximately 111 participants. Therefore, our final sample of 144 participants also met the sample size requirements for the ANCOVA method.

### 2.3. Intervention

To carry out the intervention program, the new profile of “Silver Caregiver” was implemented, with one Silver Caregiver assigned per municipality. These caregivers were trained to promote autonomy, support users in their homes, and assist in monitoring their routines. Each caregiver was assigned specific tasks, which they reported to their supervisors within the established deadlines. Their responsibilities included remote monitoring of daily tasks, establishment of routines, management of resources and incidents, functional, cognitive, social, and emotional assessment of users, intervention through accompaniment to healthcare centers, occasional support with household tasks, assistance with personal care, support in administrative procedures, provision of companionship and recreational activities, preparation of reports, creation of individual records of routines and schedules, and creation of incident reports, among others.

Each municipality was also provided with resource-monitoring equipment to oversee users’ routines. The technology applied in the project was IoT technology. This technology makes it possible to create an environment that monitors people’s activities by recording, storing, processing, and analyzing information to identify activity patterns. Its main objectives include, among others, generating new services and professionals dedicated to the care of older adults living in rural settings by implementing service models based on the use of new technologies to address isolation and loneliness; focusing on prevention and care for older adults living alone; and facilitating the provision of information to family members, guardians, or professionals responsible for the users’ condition and progress.

The technological kit included vibration, motion, and door sensors that were installed in users’ homes in locations and objects such as refrigerators, bathrooms, hallways, living rooms, kitchens, bedrooms, doors, medication pillboxes, walking aids, main entrances, and beds. For the Advanced Telecare Project of the Provincial Council of Zamora, an integrated IoT solution was implemented in the homes of participants. Sensorization devices—presence and vibration sensors manufactured by Unabiz—were installed and operated under the Sigfox (0G) low-power wide-area network (LPWAN) protocol. To ensure robust network coverage in all areas, several micro base stations (MBSs) were strategically deployed in municipal buildings across participating towns.

The caregivers’ work followed a hybrid methodology that integrated continuous remote supervision via the SilverDigi digital platform with regular in-person home visits. Continuous monitoring enabled real-time observation of users’ activity, safety, and daily routines. The system automatically detected anomalies such as inactivity, falls, or abnormal vital signs and immediately generated alerts to caregivers or authorized family members. In addition, caregivers checked the SilverDigi application at least three to four times per day—typically early in the morning and every 4–6 h thereafter—to confirm that all devices were functioning correctly and that no deviation from normal activity patterns had occurred.

Home visits were scheduled according to the individual needs and autonomy levels of each participant. As a general rule, at least one daily visit was conducted, lasting between 60 and 90 min, to verify the user’s overall condition, reinforce social and emotional support, and complement information obtained through the monitoring devices. Depending on the situation, these visits could include brief control interventions or longer sessions focused on companionship, cognitive stimulation, or assistance with household and self-care tasks.

The time interval between the first and second assessments was approximately three months, corresponding to the duration of the intervention program, including baseline evaluation, implementation phase, and post-intervention assessment.

Data collection began directly at the Unabiz sensors, which transmitted activity information (presence and vibration events) via the Sigfox network. The transmitted data were received and integrated into a cloud infrastructure hosted on Amazon Web Services (AWS). Specifically, the AWS IoT service was used for device message ingestion and management, allowing the Kwido platform (for Spain) (also hosted on AWS) to process, store, and visualize this information for social service professionals. The platform generated automated alerts to inform caregivers of potential risk situations or deviations in users’ normal routines.

The Kwido platform complies with ISO 27001 [36] and ISO 27701 [37] standards, ensuring data security and privacy. It also meets the Spanish National Security Scheme (ENS)—High Level. All AWS data centers used are located within the European Union, guaranteeing compliance with the General Data Protection Regulation (GDPR). All IoT devices were powered through the main electricity supply and equipped with battery backup for up to 24 h. The overall data flow followed a sensor → gateway → cloud architecture, with AES-256 encryption applied during data transmission and strict access control implemented at the cloud level.

Access to the collected information was strictly limited to the responsible caregivers and family members authorized by the user or their legal representative. Different access levels and permissions were established to ensure confidentiality and data protection. Both caregivers and authorized relatives could access real-time data visualization through the SilverDigi app, which remained continuously active.

The caregivers utilized the data generated by the IoT system (for Spain) through a dual supervision strategy combining real-time monitoring and periodic data analysis. Real-time monitoring enabled the immediate detection and management of critical alerts—such as falls or abnormal readings—while monthly reports compiled from the stored data allowed longitudinal analysis of behavioral patterns, functional decline, and the effectiveness of the interventions implemented. This combination of immediate supervision and periodic review optimized decision-making, improved the quality of care, and promoted a more preventive and personalized approach to supporting older adults living in rural areas.

#### IoT Alerts and Caregiver Response

In addition to subjective and clinical outcome measures, objective operational data generated by the IoT monitoring system were recorded during the intervention period. The system was configured to detect deviations from each participant’s usual daily activity patterns and to generate alerts when predefined thresholds were exceeded.

The primary alert triggers included prolonged inactivity periods (e.g., absence of detected movement beyond the expected daily routine), abnormal nighttime activity, irregular door usage patterns, and potential fall-related events detected through vibration or motion sensors. Thresholds were initially defined based on baseline observations of individual routines and were subsequently adjusted by caregivers to account for personal habits and health status.

During the three-month intervention period, a total of 150 alerts were generated by the IoT system. Of these alerts, approximately 80% resulted in an active caregiver intervention, such as a phone call, an unscheduled home visit, or coordination with family members or health services. The average response time from alert detection to caregiver action was approximately 5 min.

These alert-to-intervention events provided objective evidence of the functional role of IoT monitoring in supporting decision-making, enabling rapid responses to potential risk situations, and complementing human caregiving with continuous, real-time supervision.

The anomaly detection mechanism implemented in this study was based on a rule-based logic rather than machine learning models. Predefined thresholds were established to identify deviations from each participant’s typical daily activity patterns, using baseline observations collected prior to the intervention.

Thresholds were individualized and included criteria such as prolonged inactivity beyond the expected daily routine, abnormal nighttime movement, atypical door usage patterns, and sensor-detected events potentially associated with falls or safety risks. These thresholds were initially configured based on each participant’s habitual behavior and were subsequently refined by caregivers to minimize unnecessary alerts and to adapt to changes in health status or daily routines.

The choice of a rule-based and personalized approach was motivated by the need for transparency, interpretability, and ease of adjustment in real-world caregiving contexts, particularly in rural settings where technical support resources may be limited.

Regarding system performance, the false positive rate was not calculated as a classical algorithmic metric, as the system was designed to support human decision-making rather than autonomous event classification. However, practical system performance was assessed through the proportion of alerts that resulted in caregiver action.

During the intervention period, approximately 80% of IoT-generated alerts led to active caregiver interventions, while the remaining alerts (approximately 20%) were reviewed and classified as non-critical after verification. This proportion provides an applied proxy for the false positive burden and suggests that the system maintained a manageable alert load without overwhelming caregivers in daily practice.

### 2.4. Instruments

Based on the predefined outcome variables of the longitudinal study, sociodemographic and clinical information was collected, together with a comprehensive assessment of functional, cognitive, emotional, social, and quality-of-life domains. The level of autonomy of the users in performing ADLs and IADLs was measured, as well as their cognitive level, anxiety, depression, family functionality, perceived social support, nutritional status, life satisfaction, and quality of life (QoL), through a battery of evaluation tools. The instruments used for assessment were as follows:

Barthel Index. This scale evaluates functional status and the level of independence in patient self-care. It is applied through direct observation and assesses 10 ADLs with a score ranging from 0 to 100 points. It shows good reproducibility, with weighted kappa correlation coefficients of 0.98 intra-observer and above 0.88 inter-observer. It also has excellent internal consistency, demonstrated by a Cronbach’s alpha of 0.90 to 0.92. The established cut-off points include independence (100), slight dependence (91–99), moderate dependence (61–90), severe dependence (21–60), and total dependence (<21) [38].

Short Physical Performance Battery (SPPB). This is a simple geriatric performance test. It is a valuable tool for assessing mobility limitations. It has three sections: balance, gait, and the ability to stand up and sit down. Scores range from 0 to 12, with higher scores indicating better functional status. It has a Cronbach’s alpha of 0.86, showing good internal consistency, reliability, and validity [39].

Lawton and Brody Scale. Evaluates physical autonomy in performing IADLs. It examines 8 IADLs using a questionnaire administered either directly to the person or their caregiver. The application time is approximately 5 min and it is useful for evaluating the functional capacity of any individual. Each item is scored between 0 and 1, with a total score from 0 to 8 points, where a lower score indicates greater dependence. This questionnaire has been translated, adapted, and validated in Spanish. It demonstrates high intra- and inter-observer reproducibility (0.94) and good reliability [40,41,42].

MMSE. This is a widely used tool to assess cognitive impairment in older adults. It evaluates several cognitive areas with a score ranging from 0 to 35, with higher scores indicating better cognitive status. It has good internal consistency (Cronbach’s alpha = 0.88) and good test–retest reliability [43,44].

Charlson comorbidity index (CCI). This index, developed in the 1980s and later validated, assigns a value to each condition according to its relative risk (RR), so that comorbidities with higher RR receive a greater weighting. The age-adjusted version incorporates an additional increase based on each decade of life starting at age 50, allowing for combined age–comorbidity values. The results of the CCI and the age-adjusted CCI were subsequently classified into four categories: 0 points (no increased risk), 1–2 points (low risk), ≥3 points (high risk), and ≥5 points (very high risk) [44,45].

Yesavage Geriatric Depression Scale. This measure assesses depressive symptoms in individuals over 65 years of age. Scores are interpreted as follows: 0–5 normal, 6–9 mild depression, and >10 established depression. It demonstrates good reliability and validity [46].

Goldberg Anxiety and Depression Scale. This is a tool for evaluating depressive and anxiety symptoms in clinical populations. It consists of 18 items, with subscale scores ranging from 0 to 9, where higher scores reflect worse anxious or depressive status. Overall internal consistency is satisfactory (α = 0.80) [47,48].

APGAR Family Functionality Questionnaire. This tool assesses family functionality in older adults. It consists of 5 items scored from 0 to 3, with lower scores indicating severe dysfunction. It is a reliable and appropriate instrument (α = 0.71–0.83) [49].

Duke-UNC Functional Social Support Questionnaire. This tool evaluates perceived social support and has been validated in the Spanish population. It contains 11 items, with higher scores reflecting greater perceived support. Scores <32 indicate low perceived social support. It shows adequate internal consistency and construct validity [50].

Satisfaction With Life Scale (SWLS). This scale measures individuals’ overall judgments of satisfaction with life. It consists of 5 items rated on a 7-point Likert scale, where higher scores represent greater satisfaction. Interpretation of scores is as follows: 5–9 extremely dissatisfied, 10–14 dissatisfied, 15–19 slightly dissatisfied, 20–24 slightly satisfied, 25–29 satisfied, and 30–35 extremely satisfied. It has demonstrated high reliability and validity [51].

EuroQol-5D. This tool measures health-related quality of life (HRQoL) in the general population and in patients with different pathologies. It includes five dimensions: mobility, self-care, usual activities, pain/discomfort, and anxiety/depression. Lower scores indicate better perceived HRQoL in each dimension. Additionally, it includes a visual analog scale (VAS) from 0 to 100, with higher scores indicating the best imaginable health. It is quick to administer and provides valid and reliable results in the Spanish population [52,53].

Finally, difficulty climbing stairs was also measured using a quantitative scale from 0 to 10, where 0 represented no difficulty and 10 represented maximum difficulty.

These standardized instruments have also been widely applied in previous studies assessing technology- and IoT-based interventions among older adults. The Barthel Index and the Lawton and Brody Scale, for example, have been used in IoT-supported rehabilitation and telecare programs to monitor functional independence remotely through wearable and motion sensors [54]. Cognitive instruments such as the MMSE have been applied in smart-home and sensor-based cognitive monitoring projects to detect early cognitive decline and to evaluate digital cognitive stimulation outcomes [55].

Similarly, the Yesavage Geriatric Depression Scale and the Goldberg Anxiety and Depression Scale have been incorporated into IoT-based mental health monitoring systems to quantify depressive and anxiety symptoms and to assess the emotional impact of connected-care interventions [56,57]. Instruments addressing social and family functioning, such as the Family APGAR and the Duke-UNC scales, have also been employed in digital health programs aimed at reducing loneliness and strengthening social connectedness in community-dwelling older adults [58]. Finally, measures of life satisfaction (SWLS) and health-related quality of life (EuroQol-5D) have been recurrent outcome indicators in telecare, smart-home, and IoT-based studies that assess perceived well-being and the effectiveness of technology-assisted aging-in-place initiatives [59,60]. A summary of the variables analyzed and the respective measurement instruments is presented in Table 1.

### 2.5. Statistical Analysis

Descriptive analyses of the sociodemographic sample characteristics were performed, expressing categorical variables as absolute frequencies and percentages, and continuous variables as means and standard deviations (SDs). The normality of the dataset was tested using the Kolmogorov–Smirnov test.

Pearson correlations were performed using differential scores on the scales to establish whether comorbidity linearly influences the size of the change. To evaluate differences between the assessments conducted before and after the intervention, a paired-samples *t*-test was applied. In addition, an ANCOVA analysis was performed to observe the differences between the pre- and post-test assessments between the groups according to the variable of sex and main pathologies of the sample as a fixed factor and the pre-test scores as a covariate. The effect size was calculated in the analyses performed, considering a small effect when η^2^_p_ < 0.059, a medium effect when η^2^_p_ ≥ 0.059, and a large effect when η^2^_p_ ≥ 0.138. Statistical analyses were carried out using SPSS software, version 28 (IBM Inc., Chicago, IL, USA). Statistical significance was set at *p* < 0.05.

## 3. Results

The sample consisted of 144 older adults living in rural areas. A higher percentage of women were found in this setting, as well as a greater proportion living alone. All participants presented with some type of pathology, and their mean age was high. Table 2 below presents the sociodemographic and clinical data collected from the sample. The sociodemographic distribution of the participants reflects the typical profile of older adults living in rural areas of Castilla y León. The predominance of women (63.9%) and the high mean age (81.1 years) are consistent with demographic trends showing greater female longevity and the feminization of aging in rural Spain. The fact that two-thirds of the participants lived alone (66.7%) highlights a significant risk factor for social isolation and emotional vulnerability, which could explain the improvements observed after the intervention in variables such as social support, family functionality, and life satisfaction. The high prevalence of endocrine–metabolic (58.3%) and cardiovascular (26.4%) conditions suggests that most participants were managing chronic illnesses requiring continuous monitoring—an area where IoT-based systems and structured caregiver follow-up may have been particularly beneficial. These sociodemographic characteristics should therefore be considered when interpreting the results, as they may partly account for the magnitude of change observed in well-being and quality-of-life indicators.

The age-adjusted comorbidity index (age-adjusted CCI) showed significant correlations with several of the study variables. Specifically, moderate negative correlations were observed with the level of independence in basic activities of daily living (ADLs) assessed using the Barthel Index (r = −0.332, *p* < 0.001), with mobility limitations measured by the SPPB (r = −0.332, *p* < 0.001), with autonomy in instrumental activities of daily living (IADLs) according to the Lawton and Brody scale (r = −0.296, *p* < 0.001), and with cognitive performance assessed using the MMSE (r = −0.297, *p* < 0.001). Likewise, difficulty climbing stairs showed a significant positive correlation with the index (r = 0.321, *p* < 0.001). In other words, higher comorbidity index values were associated with lower independence in ADLs, greater frailty, reduced autonomy in IADLs, poorer cognitive performance, and greater difficulty ascending and descending stairs.

Regarding the EuroQol-5D dimensions, the comorbidity index correlated positively with the mobility dimension (r = 0.297, *p* < 0.001), as well as with the self-care (r = 0.212, *p* = 0.011) and usual activities dimensions (r = 0.210, *p* = 0.012). Thus, higher comorbidity index scores were associated with lower health-related quality of life related to mobility, self-care, and daily activities. No significant correlations were observed with depressive mood (Yesavage), anxiety and depressive symptoms (Goldberg), family functionality (APGAR), social support (Duke-UNC), life satisfaction (SWLS), the EuroQol-5D visual analog scale, or with the pain and anxiety dimensions of the EuroQol-5D (*p* > 0.05 for all) (Table 3).

After the intervention implemented with the participants, statistically significant differences were observed between the results of the first and second evaluations (Table 4) in the variables cognitive status (*p* < 0.001), depressive symptoms (*p* < 0.001), anxiety and depressive symptoms (*p* = 0.022), family functionality (*p* = 0.004), and life satisfaction (*p* < 0.001). Additionally, within HRQoL as measured by the EuroQol-5D, significant differences were found in the VAS (*p* < 0.001), pain dimension (*p* = 0.015), and anxiety dimension (*p* = 0.008). Furthermore, a significant improvement was observed in difficulty climbing stairs (*p* = 0.008). Overall, significant improvements were identified in the scores of these variables.

Beyond statistical significance, the observed changes also demonstrated clinically meaningful and behaviorally relevant improvements. The increase in cognitive status measured by the MMSE (mean change = +2.28 points) represents a moderate enhancement in cognitive performance, suggesting better attention, orientation, and short-term memory after the intervention. The reduction in depressive (Yesavage scale) and anxiety (Goldberg scale) symptoms indicates greater emotional stability and decreased psychological distress, likely reflecting the positive influence of both continuous IoT-based monitoring and regular caregiver interactions. Improvements in family functionality (APGAR) and social support (Duke-UNC) point to strengthened social connectedness and perceived support—factors that are crucial for maintaining mental health and emotional resilience in aging. Similarly, the rise in SWLS suggests a more optimistic outlook and greater sense of control in daily living. Regarding HRQoL, the increase in the EuroQol-5D VAS score (+11.5 points on average) and improvements in the pain and anxiety dimensions denote a better perception of overall health and reduced discomfort, outcomes considered clinically relevant in older adults with chronic conditions. Collectively, these findings reveal that the combined Silver Caregiver and IoT intervention not only produced statistically significant outcomes but also generated tangible improvements in cognitive, emotional, and functional well-being, contributing to greater autonomy, safety, and quality of life among participants. While no statistically significant improvements were observed in objective physical function measures, functional status was maintained over the follow-up period.

An ANCOVA analysis was performed with the significant variables from the previous analysis, with gender of participants as a fixed factor and pre-test score as a covariate. Statistically significant differences were observed between pre- and post-intervention assessments in the pain dimension of the EQ-5D scale (*p* = 0.020). Specifically, male participants reported a greater reduction in perceived pain compared with females, suggesting that sex may influence the pain-related outcomes following the intervention. This result indicates that men experienced better post-intervention improvement in pain perception, possibly related to differences in baseline characteristics or responsiveness to the combined Silver Caregiver and IoT program. Table 5 presents these results in detail.

Table 6 presents the results of the ANCOVA analysis conducted exclusively on the variables that showed statistical significance in Table 3, in order to improve clarity and avoid an excessively large and difficult-to-interpret table. The analysis included social support (Duke-UNC), life satisfaction (SWLS), and health-related quality of life (HRQoL, VAS of the EuroQol-5D), taking the main pathology as a fixed factor and the pre-test score as a covariate.

The results revealed statistically significant differences between pathological groups in the three variables considered: social support (*p* = 0.029, η^2^_p_ = 0.07), life satisfaction (*p* = 0.004, η^2^_p_ = 0.077), and QOL (*p* = 0.016, η^2^_p_ = 0.058). According to the η^2^_p_ values, all effects showed a medium magnitude, indicating a moderate impact of the main pathology on the evolution of these dimensions.

Specifically, patients with dementia/Parkinson’s and other conditions showed greater increases in social support and life satisfaction, whereas the most notable improvements in QoL were observed among the endocrine–metabolic groups. In contrast, participants with dementia/Parkinson’s disease and others exhibited less improvement in perceived QoL.

Subsequently, post hoc analyses were conducted to explore the specific differences between pathological groups (Table 7). The results revealed that the most substantial improvements in social support occurred among patients with dementia/Parkinson’s disease and others (*p* = 0.011), followed by those with endocrine–metabolic disorders (*p* = 0.047). Similarly, in life satisfaction, significant increases were observed in patients with dementia/Parkinson’s disease and others (*p* < 0.001).

Regarding HRQoL, measured by the EuroQol-5D scale, significant improvements were found in the dementia/Parkinson’s and endocrine–metabolic (*p* = 0.009) groups.

In addition to the questionnaire-based outcomes, objective system-level indicators supported the contribution of IoT monitoring to the intervention. Over the three-month follow-up period, the IoT system generated 150 alerts related to deviations from participants’ normal activity patterns. The majority of these alerts (approximately 80%) triggered direct caregiver actions, with a mean response time of 5 min, indicating effective integration between technological monitoring and human intervention.

## 4. Discussion

The aim of this study was to maintain the capacities of older adults living in rural areas through care and digitalization with IoT technology in order to ensure safety and autonomy in their homes and to provide vital emotional support in the final stage of life.

The use of new technologies, such as IoT technology, enables older adults to enhance and enrich their individual and social development, while at the same time optimizing their quality of life from technical, economic, political, and cultural perspectives [61]. Previous research has shown that assistive tools and new technologies can play a crucial role in this area. However, their specificity is limited due to the constant change, development, and updating of technology [62,63].

The results of the correlational analysis show that a higher level of age-adjusted comorbidity is primarily associated with poorer physical functioning, reduced autonomy in basic and instrumental activities of daily living, lower cognitive performance, and greater mobility difficulties, as evidenced by the positive correlation with difficulty climbing stairs. Likewise, the positive relationship between the comorbidity index and the EuroQol-5D dimensions of mobility, self-care, and usual activities reinforces the notion that individuals with a higher burden of comorbidities perceive a lower quality of life in domains closely linked to physical functioning. Overall, these findings are consistent with previous literature describing comorbidity as a key factor in functional deterioration, frailty, and cognitive decline in older adults, and they underscore the need to design interventions that integrate continuous monitoring and support for autonomy to mitigate these effects [64].

Our results show that more than half of the rural population is composed of women, with an average age of over 80 years, most of whom live alone. Cardiovascular and metabolic/endocrine conditions were the predominant pathologies. Statistically significant differences were also observed after the intervention involving care and the application of IoT technology, particularly in cognitive status, depressive symptoms, anxiety symptoms, family functionality, life satisfaction, HRQoL (specifically in the pain dimension, anxiety dimension, and VAS), and difficulty climbing stairs. All variables improved, thus achieving positive outcomes across all measures. In line with our findings, recent evidence supports the use of sensor- and IoT-based interventions in older populations. For example, a randomized clinical trial of wearable sensor-based interactive cognitive-motor training demonstrated a significant cognitive improvement in older adults (mean increase ~1.94 points; *p* < 0.05) [65]. Moreover, a systematic review and meta-analysis found that sensor-based technologies with biofeedback were more effective than traditional physical exercise in improving gait, balance, and functional performance in older adults [66].

The assessment of HRQoL provides a comprehensive view of the diverse aspects that influence well-being during aging. In this context, HRQoL is understood as a multifaceted process that encompasses health status, mobility, self-care, usual activities, pain/discomfort, and anxiety/depression. However, older adults often prioritize maintaining functional capacity and avoiding disabling diseases in order to experience healthy aging and a good quality of life. HRQoL has become one of the most important indicators in social and health interventions, particularly in the field of primary healthcare [67,68]. Importantly, the absence of statistically significant changes in objective physical function measures such as the Barthel Index and the SPPB should not be interpreted as a lack of intervention effectiveness. In gerontological research, maintaining functional status over time—particularly in very old populations with high comorbidity—is considered a positive and clinically meaningful outcome. Preventing further decline in basic activities of daily living and mobility represents a relevant achievement, especially in rural contexts where access to rehabilitation and healthcare services is limited.

The results show statistically significant differences between the pre- and post-test in HRQoL, specifically in the pain dimension, when sex was considered as a covariate. In this case, men reported a better perception of their HRQoL in the pain dimension. Particularly relevant is the observation that gender differences influence the perception of HRQoL regarding pain. This highlights the need to consider gender differences when designing health interventions for older adults. In general terms, findings from several studies confirm that women experience aging with a lower HRQoL. Women have undergone a more complex life course, both physiologically [69] and socially, due to the repercussions associated with multiple caregiving responsibilities [70]. Nonetheless, another study also indicates that mental health problems are more common among older women, as evidenced by both national [71] and international research [72]. In terms of objective health, a higher prevalence of health problems has been observed among women.

Statistically significant differences were also found between pre-test and post-test scores when considering participants’ main pathology. Specifically, significant differences were observed in social support between those with cardiovascular/stroke conditions and those with dementia/Parkinson’s disease, pulmonary disease, or metabolic/endocrine conditions. All of these groups showed greater social support compared to participants with cardiovascular/stroke conditions. Regarding life satisfaction, participants with cardiovascular/stroke conditions reported being slightly dissatisfied after the program, while those with dementia/Parkinson’s and metabolic/endocrine conditions reported being slightly satisfied, and those with pulmonary disease reported being satisfied. Nevertheless, all groups improved their life satisfaction scores after the intervention. Finally, statistically significant differences were obtained after the intervention in HRQoL, as measured by the VAS. Participants with pulmonary disease showed the highest HRQoL scores, followed by those with metabolic/endocrine, cardiovascular/stroke, and lastly dementia/Parkinson’s conditions. All groups improved their HRQoL scores after the intervention. These findings underscore the importance of tailoring interventions to the specific needs of each patient group.

So far, no studies have reported an intervention similar to the one presented here. However, it has been shown that individuals with various pathologies often experience a decline in HRQoL due to physical or cognitive limitations, anxiety, emotional challenges, or uncertainty. Social support can help mitigate these negative effects and is essential in the process [73,74,75]. Life satisfaction often improves when patients feel accompanied and understood by both their loved ones and healthcare professionals [76,77,78,79]. In this sense, IoT technology and home care can help monitor patient safety at home, facilitate communication and symptom follow-up, provide continuous monitoring and alerts, track health data, and/or deliver personalized self-care reminders, thereby offering greater reassurance and improving some of these aspects [80,81].

Studies focusing on smart-home IoT systems report high feasibility and positive acceptability among older adults living independently, combining environmental and motion sensors to support aging-in-place [82]. As highlighted by Chen et al. [82], connected health approaches are reshaping the delivery of care, making it ‘more proactive, more preventive, and more precisely targeted and, thus, more effective’; remote support and smart services have demonstrated substantial value in addressing the challenges posed by aging populations and chronic diseases.

One of the key contributions of this study is the demonstration of significant improvements in various measures of well-being and mental and physical health after implementing the intervention, which combines traditional care with IoT technology. These improvements were observed in variables such as cognitive status, depressive and anxiety symptoms, family functioning, life satisfaction, and HRQoL.

The evidence generated in this study supports the integration of IoT devices and telemonitoring within routine healthcare policies, offering actionable models for remote assessment, prevention, and personalized intervention among older adults. Policymakers can use these results to justify investment in digital infrastructure and support programs enabling independent living and early detection of risk situations in rural contexts. These findings highlight the need to update health policy frameworks to include scalable digital health interventions that complement conventional care, reduce operational costs, and improve access and continuity for older populations living in underserved rural areas. From a technical perspective, the high proportion of actionable alerts highlights the suitability of a personalized rule-based anomaly detection approach for applied IoT caregiving systems, where interpretability and caregiver acceptance are critical factors.

These sensor-driven approaches thus provide a stronger basis for our assertion that the combined caregiver–IoT intervention can effect meaningful changes across cognitive status, depressive/anxiety symptoms, life satisfaction, and health-related quality of life. Nonetheless, given the relative novelty of full IoT-sensor-based caregiving paradigms in rural older populations, further empirical studies remain necessary. Although the study design does not allow for a complete separation of the effects of human caregiving and IoT monitoring, the inclusion of objective alert-to-intervention data strengthens the evidence for the technical contribution of the IoT component. The high proportion of alerts leading to rapid caregiver actions demonstrates that the technology functioned as an active trigger for timely interventions, rather than as a passive background system. This real-time responsiveness represents an added value beyond traditional scheduled visits, particularly in rural contexts where immediate access to services is limited.

As study limitations, it should be noted that the sample size may be limited, which could affect the ability to generalize the results to a broader population of older adults living in rural settings. Likewise, another important limitation of the study is that the sample was recruited only in a small geographic region, which further limits the generalizability of the results to other rural areas with different demographic or contextual characteristics. The follow-up period may also be relatively short compared to the chronic and progressive nature of many diseases in older adults. Furthermore, the study design may present inherent limitations, such as the absence of a randomized control group or the presence of selection or information biases, which may affect internal validity and the interpretation of results. In addition, it was not possible to clearly distinguish the specific effects of the Silver Caregiver’s manual interventions from those attributable to IoT-based monitoring, as both components were implemented simultaneously. Additionally, the use of some subjective measures could introduce biases in the interpretation of the findings.

## 5. Conclusions

The results of this study suggest that the combined IoT-based monitoring and Silver Caregiver intervention does not primarily lead to measurable improvements in objective physical function over the short term but rather contributes to sustaining basic functional abilities while significantly enhancing psychological well-being, social connectedness, and perceived quality of life in older adults living in rural settings. It is crucial to take these differences into account when designing health and care interventions tailored to each group in order to maximize their effectiveness and appropriateness to individual needs. In addition, the use of IoT technology in the care of older adults in rural areas emerges as a promising tool to enhance safety, autonomy, and HRQoL in this population. Its integration into care interventions can offer innovative and effective solutions to address the specific challenges faced by older adults in rural environments. From a gerontological perspective, sustaining physical function while improving emotional and social dimensions represents a meaningful outcome that may help delay functional decline and support aging in place.

## Figures and Tables

**Table 1:** Variables and instruments.

Instrument	Variable
Barthel	Functional status, level of independence for ADLs
SPPB	Mobility limitations
Lawton y Brody	Autonomy for IADLs
MMSE	Cognitive status
CCI	Comorbidity
Yesavage	Depressive symptoms
Goldberg	Anxiety and depressive symptoms
Apgar	Family functionality
Duke-Unc	Social support
SWLS	Life satisfaction
EuroQol-5D	HRQoL

ADLs: activities of daily living; SPPB: Short Physical Performance Battery; IADLs: instrumental activities of daily living; MMSE: Mini-Mental State Examination; CCI: Charlson comorbidity index; SWLS: Satisfaction With Life Scale; HRQoL: health-related quality of life.

**Table 2:** Sociodemographic and clinical data.

Variables		*N* (144)	%
Sex	Male	52	36.1
	Female	92	63.9
Age		81.10 ± 7.712 (44–94)	
Principal pathology	Cardiovascular-stroke	38	26.4
	Dementia–Parkinson’s disease and others (pulmonary or cancer)	22	9.0
	Endocrine–Metabolic	84	58.3
Civil status	Married	31	21.5
	Single	28	19.4
	Widowed	85	59
Currently lives	Living alone	96	66.7
	Living with a family member or caregiver	48	33.4
Home has stairs	Yes	88	61.1
	No	56	38.9

**Table 3:** Pearson correlation between study variables and age-adjusted CCI.

Variables (Instruments)	Age-Adjusted CCI (N = 144)	
	r	*p*
Functional status, level of independence for ADLs (Barthel)	−0.332 **	<0.001
Mobility limitations (SPPB)	−0.332 **	<0.001
Autonomy for IADLs (Lawton and Brody)	−0.296 **	<0.001
Cognitive status (MMSE)	−0.297 **	<0.001
Depressive symptoms (Yesavage)	0.101	0.229
Anxiety and depressive symptoms (Goldberg)	−0.014	0.868
Family functionality (APGAR)	0.086	0.305
Social support (Duke-UNC)	−0.046	0.586
Life satisfaction (SWLS)	0.007	0.931
HRQoL, VAS (EuroQol-5D)	−0.069	0.409
Mobility dimension (EuroQol-5D)	0.297 **	<0.001
Self-care dimension (EuroQol-5D)	0.212 *	0.011
Usual activities dimension (EuroQol-5D)	0.210 *	0.012
Pain dimension (EuroQol-5D)	0.132	0.115
Anxiety dimension (EuroQol-5D)	−0.032	0.704
Difficulty climbing stairs	0.321 **	<0.001

SD: standard deviation; CI: confidence interval; ADLs: activities of daily living; SPPB: Short Physical Performance Battery; IADLs: instrumental activities of daily living; MMSE: Mini-Mental State Examination; SWLS: Satisfaction With Life Scale; health-related quality of life (HRQoL); VAS: visual analog scale. * *p* < 0.005; ** *p* < 0.01.

**Table 4:** Paired-samples *t*-test between pre- and post-intervention scores.

Variables (Instruments)	Pre-Test (Mean ± SD)	Post-Test (Mean ± SD)	Differential Score (Mean ± SD)	CI 95%		*p*
				LI	LS	
Functional status, level of independence for ADLs (Barthel)	91.44 ± 17.177	90.85 ± 21.049	0.590 ± 11.023	−1.225	2.406	0.522
Mobility limitations (SPPB)	5.97 ± 3.534	5.99 ± 3.837	−0.021 ± 2.787	−0.480	0.438	0.929
Autonomy for IADLs (Lawton and Brody)	6.13 ± 1.997	6.58 ± 2.749	−0.451 ± 2.781	−0.909	0.007	0.721
Cognitive status (MMSE)	28.35 ± 6.684	30.63 ± 6.058	−2.278 ± 5.189	−0.345	0.498	<0.001 **
Depressive symptoms (Yesavage)	4.03 ± 3.319	2.91 ± 1.138	1.125 ± 2.838	−3.133	−1.423	<0.001 **
Anxiety and depressive symptoms (Goldberg)	5.20 ± 4.598	4.22 ± 5.289	0.979 ± 5.063	0.145	1.813	0.022 *
Family functionality (APGAR)	8.63 ± 3.553	9.31 ± 1.756	−0.681 ± 2.825	−1.146	−0.215	0.004 **
Social support (Duke-UNC)	43.72 ± 9.946	46.55 ± 8.973	−2.823 ± 9.198	−1.053	−5.272	<0.001 **
Life satisfaction (SWLS)	18.23 ± 4.424	19.92 ± 4.694	−1.684 ± 3.833	−2.314	−1.053	<0.001 **
HRQoL, VAS (EuroQol-5D)	59.65 ± 20.532	71.18 ± 21.629	−11.528 ± 22.789	−15.282	−7.774	<0.001 **
Mobility dimension (EuroQol-5D)	1.47 ± 0.528	1.47 ± 0.541	0.001 ± 0.515	−0.085	0.85	1.000
Self-care dimension (EuroQol-5D)	1.27 ± 0.505	1.22 ± 0.465	0.049 ± 0.448	−0.025	0.122	0.195
Usual activities dimension (EuroQol-5D)	1.39 ± 0.543	1.38 ± 0.613	0.014 ± 0.567	−0.080	0.107	0.769
Pain dimension (EuroQol-5D)	1.73 ± 0.594	1.61 ± 0.593	0.118 ± 0.573	0.024	0.212	0.015 *
Anxiety dimension (EuroQol-5D)	1.24 ± 0.446	1.15 ± 0.392	0.097 ± 0.432	0.026	0.168	0.008 **
Difficulty climbing stairs	5.22 ± 3.424	4.51 ± 3.530	0.708 ± 3.150	0.189	1.227	0.008 **

SD: standard deviation; CI: confidence interval; ADLs: activities of daily living; SPPB: Short Physical Performance Battery; IADLs: instrumental activities of daily living; MMSE: Mini-Mental State Examination; SWLS: Satisfaction With Life Scale; health-related quality of life (HRQoL); VAS: visual analog scale. * *p* < 0.005; ** *p* < 0.01.

**Table 5:** ANCOVA analysis of cognitive status according to participant gender.

Variables	Group	Pre-Test (Mean ± SD)	Post-Test (Mean ± SD)	Differential Score (Mean ± SD)	*p*	η^2^_p_	95% CI	
							LI	LS
Pain dimension (EuroQol-5D)	Female	1.79 ± 0.584	1.72 ± 0.599	0.204 ± 0.087	0.020 *	0.038	0.032	0.376
	Male	1.62 ± 0.599	1.42 ± 0.537	−0.204 ± 0.087			−0.376	−0.032

MMSE: Mini-Mental State Examination. * *p* < 0.005; Covariate: pre-test score.

**Table 6:** Post hoc test of the ANCOVA analysis of significant variables according to the participants’ main pathology.

Variables	F	gl	*p*	η^2^_p_	Principal Pathology (N)	LS (Mean ± SD)	95% CI	
							LI	LS
Social Support (Duke-Unc)	3.623	2, 140	0.029 *	0.07	Cardiovascular/Stroke (38)	0.30 ± 1.224	−2.11	2.72
					Dementia–Parkinson’s disease and others (22)	5.45 ± 1.598	2.29	8.61
					Endocrine–Metabolic (84)	3.27 ± 0.821	1.65	4.89
Life Satisfaction (SWLS)	5.878	2, 140	0.004 *	0.077	Cardiovascular/Stroke	0.61 ± 0.575	−0.52	1.74
					Dementia–Parkinson’s disease and others	3.78 ± 0.746	2.31	5.26
HRQoL, VAS (Euro-Qol-5D)	4.275	2, 140	0.016 **	0.058	Cardiovascular/Stroke	8.14 ± 3.176	1.86	14.42
					Dementia–Parkinson’s disease and others	3.00 ± 4.11	−5.12	11.14
					Endocrine–Metabolic	15.29 ± 2.11	11.10	19.47

SWLS: Satisfaction with Life Scale; health-related quality of life (HRQoL); VAS: visual analog scale. * *p* < 0.005; ** *p* < 0.01. Covariate: pre-test score.

**Table 7:** ANCOVA analysis of significant variables according to the participant’s main pathology.

Variables	Principal Pathology	Pre-Test (Mean ± SD)	Post-Test (Mean ± SD)	Principal Pathology	Pre-Test (Mean ± SD)	Post-Test (Mean ± SD)	Differential Score (Mean ± SD)	*p*	95% CI	
									LI	LS
Social Support (Duke-Unc)	Cardiovascular/Stroke	41.39 ± 9.351	42.95 ± 9.992	Dementia–Parkinson’s disease and others	42.95 ± 11.914	48.82 ± 7.281	−5.14 ± 2.009	0.011 *	−9.12	−1.17
				Endocrine–Metabolic	44.98 ± 9.555	47.58 ± 8.511	−2.97 ± 1.482	0.047 *	−5.90	−0.04
Life Satisfaction (SWLS)	Cardiovascular/Stroke	16.55 ± 4.144	17.68 ± 4.497	Dementia–Parkinson’s disease and others	16.82 ± 4.205	21.05 ± 5.047	−3.18 ± 0.930	<0.001 **	−5.01	−1.34
HRQoL, VAS (EuroQol-5D)	Cardiovascular/Stroke	52.89 ± 17.228	65.00 ± 18.995	Endocrine–Metabolic	62.38 ± 21.432	76.07 ± 20.061	−12.28 ± 4.622	0.009 *	−21.41	−3.14

SWLS: Satisfaction with Life Scale; health-related quality of life (HRQoL); VAS: visual analog scale. * *p* < 0.005; ** *p* < 0.01. Covariate: pre-test score.

## Data Availability

Data for this research are available upon request from the corresponding author.
